# Real-Life Use of Filgotinib in Rheumatoid Arthritis: A Retrospective Cohort Study

**DOI:** 10.3390/jcm13237185

**Published:** 2024-11-27

**Authors:** Vincenzo Raimondo, Maurizio Caminiti, Domenico Olivo, Pietro Gigliotti, Massimo L’Andolina, Pietro Muto, Roberta Pellegrini, Giuseppe Varcasia, Caterina Bruno, Laura Massaro, Giuseppa Pagano Mariano, Jessica Maria Elisa Luppino, Mariateresa Cirillo, Virginia Caira, Marilena Calabria, Jacopo Ciaffi, Clodoveo Ferri, Francesco Ursini

**Affiliations:** 1 Rheumatology Clinic, ASP Crotone, 88836 Crotone, Italy; 2 Rheumatology Unit, Grande Ospedale Metropolitano, 89124 Reggio Calabria, Italy; mauriziocaminiti@tin.it (M.C.); giusypaganomariano@libero.it (G.P.M.); 3 Rheumatology Outpatient Clinic, ASP Crotone, Via Nazione Unite, 88900 Crotone, Italy; olivod@libero.it; 4 Rheumatology Clinic, ASP Cosenza, 87100 Cosenza, Italy; pietrogigliotti1@virgilio.it; 5 Rheumatology Outpatient Clinic, ASP Vibo Valentia-Tropea Hospital, 89861 Tropea, Italy; mxland@libero.it (M.L.); mteresacirillo@gmail.com (M.C.); 6 Internal Medicine Unit, Hospital of Paola-Cetraro, 87027 Paola, Italy; pietro.muto87@gmail.com; 7 Internal Medicine Unit “M. Valentini”, Hospital of Cosenza, 87100 Cosenza, Italy; r.pellegrini62@libero.it; 8 Rheumatology Unit, Castrovillari Hospital, 87012 Castrovillari, Italy; g.varcasia@virgilio.it (G.V.); virginiacaira@gmail.com (V.C.); 9 Internal Medicine Unit, AOU “Dulbecco”, 88100 Catanzaro, Italy; caterina-bruno@libero.it (C.B.); marilena.calabria.1982@gmail.com (M.C.); 10 Rheumatology Clinic, “Distretto Tirreno”-ASP Cosenza, 71020 Cosenza, Italy; laura.massaro84@gmail.com; 11 Rheumatology Clinic, “Madonna dello Scoglio” Hospital, 88836 Cotronei, Italy; luppinojessica@gmail.com (J.M.E.L.); clferri@unimore.it (C.F.); 12 Medicine & Rheumatology Unit, IRCCS Istituto Ortopedico Rizzoli, 40136 Bologna, Italy; jacopo.ciaffi@ior.it (J.C.); francesco.ursini@ior.it (F.U.); 13 Department of Biomedical and Neuromotor Sciences (DIBINEM), Alma Mater Studiorum University of Bologna, 40126 Bologna, Italy

**Keywords:** rheumatoid arthritis, JAK inhibitors, filgotinib

## Abstract

**Background:** Janus kinase inhibitors (JAKis) are a novel class of drugs interfering with intracellular signaling of type I and type II cytokines, which play a crucial role in immune dysregulation associated with several chronic inflammatory diseases. Filgotinib (FIL), in particular, is the newest member of the JAKi class and exerts its therapeutic effects by selectively targeting and inhibiting the kinase activity of JAK1. While the efficacy of FIL in rheumatoid arthritis (RA) has been confirmed in clinical trials, real-world evidence may provide better insights into its effectiveness and safety in routine clinical practice. **Methods:** We performed a multicenter, retrospective cohort study investigating the real-life effectiveness and safety of FIL in adult patients with RA. Demographic information, disease characteristics, prior treatment history, and comorbid conditions were retrieved from clinical records at baseline (M0) and after 3 (M3) and 6 months (M6) of treatment. **Results:** A total of 82 patients (63 women) agreed to participate in the study, of whom 39 (47.6%) were older than 65 years. The average RA duration was 13 ± 9 years; 19 patients (23.1%) were current or former smokers, and 4 patients (4.9%) had a history of cardiovascular events. Most patients had previously received at least one biologic disease-modifying antirheumatic drug (range: 1–6+); in addition, 11 patients (13.4%) had been already exposed to another JAKi. During the follow-up, 7 patients discontinued treatment due to primary failure (*n* = 3) or adverse events (*n* = 4). Significant reductions in pain and number of tender and swollen joints were observed at M3 and M6. A relevant proportion of patients achieved DAS28-CRP remission at M3 and M6 (46.3% and 66.2%, respectively). **Conclusions:** Our data provide additional insight into the effectiveness of filgotinib in a real-world setting, even among patients with difficult-to-treat RA and a high prevalence of cardiovascular risk factors.

## 1. Introduction

Rheumatoid arthritis (RA) is a chronic inflammatory autoimmune disease characterized by synovitis, tenosynovitis, and extra-articular manifestations, which can potentially lead to joint damage, disability, poor quality of life, and an increased risk of cardiovascular diseases [1]. Given the immune dysregulation underlying the disease, the available therapies are based on immunomodulatory drugs such as conventional synthetic disease-modifying antirheumatic drugs (DMARDs) (e.g., methotrexate) or biologic DMARDs targeting key cytokines (e.g., TNF inhibitors, IL-6 inhibitors) or cells (e.g., the selective T-cell costimulation modulator abatacept of the B-cell-depleting agent rituximab) involved in RA immunopathogenesis [2].

Janus kinase inhibitors (JAKis) are a novel class of DMARDs (i.e., targeted synthetic DMARDs (tsDMARDs)) that have revolutionized the landscape of autoimmune and inflammatory disease management [3]. Acting as intracellular signaling modulators, these molecules intervene in the Janus kinase–signal transducer and activator of transcription (JAK-STAT) pathway, a crucial cascade involved in the transmission of signals from type I and type II cell surface receptors to the nucleus [4]. This pathway is central to the regulation of immune responses, making JAKis a targeted and effective therapeutic strategy for diseases characterized by dysregulated immunity such as rheumatoid arthritis (RA), psoriasis/psoriatic arthritis (Pso/PsA), and ulcerative colitis (UC) [5]. Compared with biologic DMARDs (bDMARDs), the versatility of JAKis lies in their ability to selectively block specific JAK isoforms, including JAK1, JAK2, JAK3, and TYK2, thereby disrupting the signaling pathways mediated by different cytokines [6], with the overarching goal to modulate immune responses without causing global immunosuppression.

**Figure 1:**
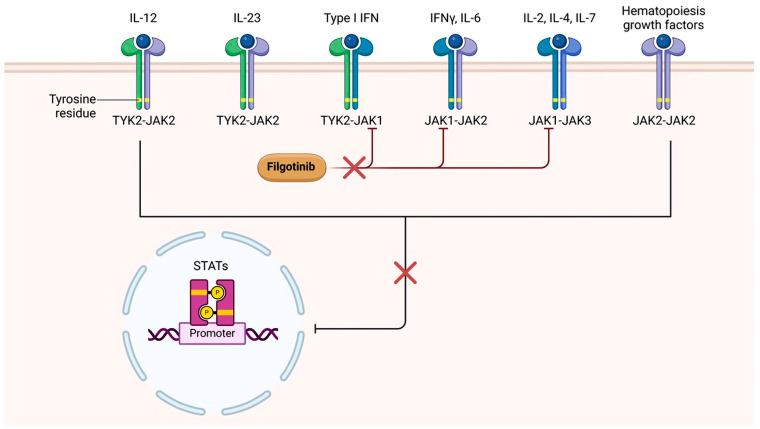
Mechanism of action of filgotinib. Different from pan-JAKi, such as tofacitinib and baricitinib, filgotinib selectively inhibits the activity of JAK1, thereby blocking the intracellular signaling pathways of pro-inflammatory cytokines crucial to the pathogenesis of RA, such as IL-6 and IFNs. This inhibition blocks the migration of STATs into the nucleus, thereby impeding the transcription of genes that activate inflammatory mechanisms. Created in BioRender. Ciaffi, J. (2024). https://BioRender.com/m21v977.

Filgotinib—the newest member of the JAKi class—exerts its therapeutic effects by selectively targeting and inhibiting the kinase activity of JAK1 [7], disrupting the intracellular signaling cascade initiated by key cytokines (e.g., interleukin-6 (IL-6) and type-I interferons (IFN1), which are integral to the inflammatory processes involved in the pathophysiology of RA (Figure 1). Compared to non-selective JAKis, the targeted approach of filgotinib may theoretically allow for a more precise interference with inflammatory pathways while minimizing the impact on other JAK isoforms; this has been translated into a favorable efficacy and safety profile, as already demonstrated in randomized clinical trials (RCTs) and long-term extension (LTE) studies [8,9,10,11,12]. However, it is now recognized that real-world evidence (RWE) plays a critical role in complementing findings from RCTs by providing insights into the performance of pharmaceutical interventions in everyday clinical practice. This concept is even more emphasized when applied to JAKis, particularly in the aftermath of the ORAL Surveillance trial [13], a post-marketing safety study that failed to demonstrate non-inferiority of tofacitinib compared with TNF inhibitors (TNFis) with regard to the risk of major cardiovascular events (MACEs) and cancer. This trial raised safety concerns, prompting the European Medicines Agency (EMA) to issue recommendations aimed at minimizing the risk of serious side effects and to restrict their use in patients identified as being at higher risk, including those aged 65 years or older, individuals with an increased risk of major cardiovascular issues, smokers, and those with a heightened risk of cancer. On the other hand, massive RWE data provided robust reassuring results [14,15,16]. Unfortunately, a similar amount of data is not yet available for other members of the JAKi class, including filgotinib.

On this basis, the aim of our study was to provide additional evidence on the effectiveness and safety of filgotinib in real-world patients with RA. 

## 2. Methods

### 2.1. Study Design

A multicenter, retrospective cohort study to evaluate the real-life effectiveness and safety of filgotinib in adult patients with RA.

### 2.2. Participants

Consecutive patients aged 18 years and older diagnosed with RA and starting FIL during the study period (January 2021–December 2021) were retrospectively recruited from 11 rheumatology clinics distributed across the Calabria Region (Southern Italy). According to a previous study from the same research group integrating administrative data from the institutional payer [17], these centers account for >90% of total bDMARDs (and tsDMARDs) prescriptions for the rheumatology area (Calabria Region).

Of note, we deliberately included only patients who initiated FIL therapy before January 2022, prior to the publication of the results of the ORAL Surveillance study [13].

This decision was prompted by the recognition that the emergence of new clinical data and updated recommendations from the regulatory authorities had a significant impact on physicians’ prescribing behaviors. By incorporating patients treated before this milestone, we aimed to capture a broader picture of filgotinib use in clinical practice, reflecting the temporal context in which prescriptions were made and mitigating the risk of an immediate influence of new evidence on patient management.

As per prescribing recommendations relevant to that period, FIL was prescribed to patients with moderate to severe RA who had responded inadequately to or who were intolerant to one or more DMARDs.

### 2.3. Data Collection

Baseline data, including demographic information, disease characteristics, prior treatment history, and comorbid conditions (with special focus on MACE risk factors—smoking, history of atherosclerotic cardiovascular disease (ASCVD), obesity, high blood pressure, dyslipidemia, and diabetes) were collected. Similarly, clinical assessments and laboratory results were retrieved from the clinical records of individual follow-up visits at baseline (M0, before starting filgotinib), at 3 ± 1 months (M3), and at 6 ± 1 months (M6). According to local practice, a core set of outcome measures is required to complete the prescription form for bDMARDs and tsDMARDs; therefore, these measures were available for all patients. The disease activity score, including 28 joints and C-reactive protein (DAS28-CRP) [18], and the simplified disease activity index (SDAI) [19] were used to define disease states as follows: (a) remission (DAS28-CRP < 2.6 or SDAI ≤ 3.3), (b) low disease activity (DAS28-CRP ≥ 2.6 and ≤3.1 or SDAI > 3.3 or ≤11), (c) moderate disease activity (DAS28-CRP ≥ 3.1 and ≤5.1 or SDAI > 11 and ≤26), or (d) high disease activity (DAS28-CRP > 5.1 or SDAI > 26). A 10 cm visual analog scale (VAS) was used to prospectively assess pain. Adverse events (AEs) were systematically recorded at each follow-up visit.

### 2.4. Ethical Approval

The study protocol was approved by the local Ethics Committee (Comitato Etico Territoriale—Regione Calabria, Italy), protocol number 70 (9 November 2023). Informed consent was obtained from all patients at the time of enrollment. All procedures were performed in accordance with the 1964 Declaration of Helsinki and its later amendments.

### 2.5. Data Analysis

Data are expressed as the mean ± standard deviation (SD), median (range), or number (percentage), as appropriate. Student’s *t*-tests and Mann–Whitney U tests were used to compare differences between normally and non-normally distributed continuous variables, respectively. Fisher’s exact test was used to compare categorical variables. A *p*-value of <0.05 was considered statistically significant. All analyses were performed using the Statistical Package for Social Sciences (SPSS) software version 26.0 (IBM, Armonk, NY, USA).

## 3. Results

### 3.1. Baseline Characteristics

During the recruitment period, a total of 101 patients started treatment with FIL for inadequate control of disease activity or steroid dependence. All patients were treated with FIL 200 mg once daily as the study recruited patients treated before the EMA’s safety committee recommended minimizing the risk of serious side effects associated with JAKi [20].

**Figure 2:**
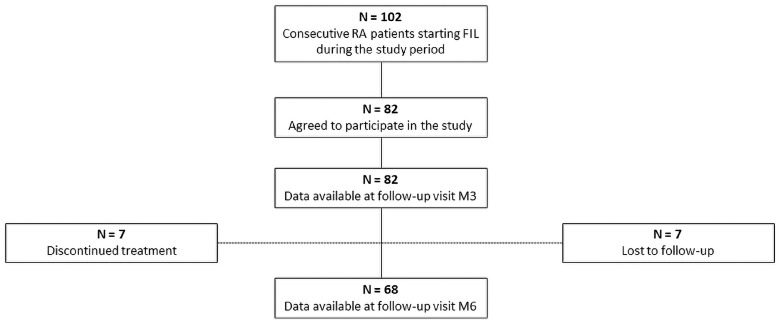
Baseline characteristics: enrollment of patients and follow-up.

Of these, 82 (63 females) agreed to participate in the study and signed the informed consent form (Figure 2). The general characteristics of the study population are detailed in Table 1. The mean age was 62 ± 13 years; notably, 39 patients (47.6%) were older than 65 years. The average disease duration was 13 ± 9 years. Regarding other cardiovascular risk factors, 19 patients (23.1%) were current or former smokers, 4 patients had a history of ASCVD, 12 patients (14.6) had diabetes, 43 patients (52.4%) had high blood pressure, and 28 patients (34.1%) had dyslipidemia. Most patients (61%) received at least one bDMARD (range 1–6) and 18 (22%) received three or more bDMARDs; furthermore, 11 patients (13.4%) had been already exposed to another JAKi. Filgotinib was administered as monotherapy in 57 patients (69.5%).

### 3.2. Effectiveness of Filgotinib in Real Life

The number of patients available for analysis at each follow-up visit is reported in Figure 2. Seven patients did not attend the M6 visit and thus were lost to follow-up; seven patients discontinued treatment after the M3 follow-up visit because of primary failure (n = 3) or adverse events (nausea, n = 1; diplopia, n = 1; malaise, n = 1; recent diagnosis of metastatic cancer, n = 1). Accordingly, a total of 82 patients were available for analysis at M3 and 68 at M6.

**Figure 3:**
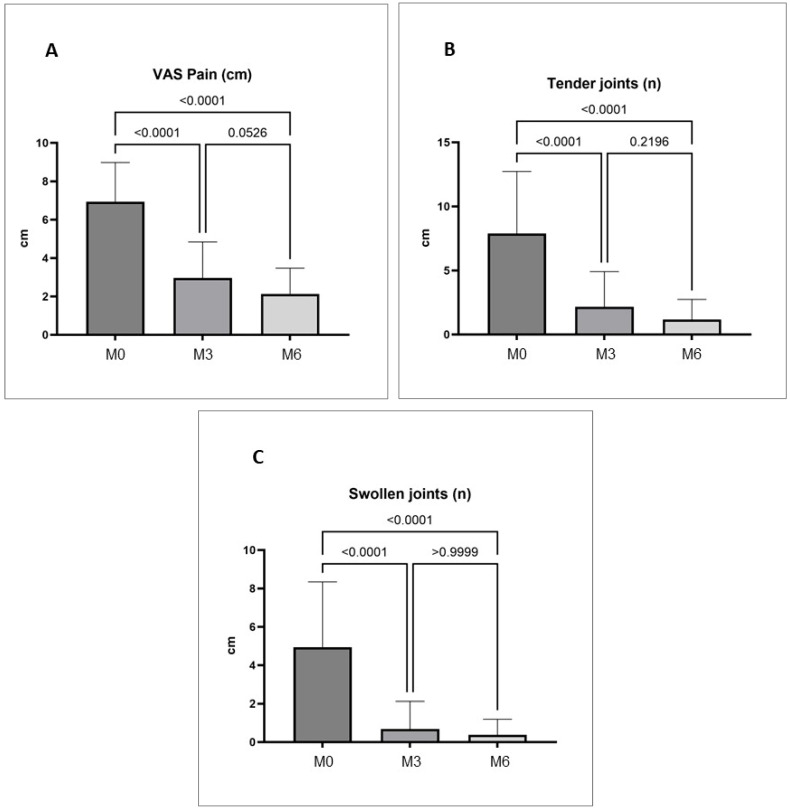
(**A**) Change in VAS pain at follow-up compared to baseline. (**B**) Evaluation of tender joints at follow-up compared to baseline. (**C**) Evaluation of swollen joints at follow-up compared to baseline.

A significant reduction in VAS pain, TJC, and SJC was observed at month three (M3) and month six (M6) compared with baseline values (M0), as reported in Table 2 and Figure 3.

**Figure 4:**
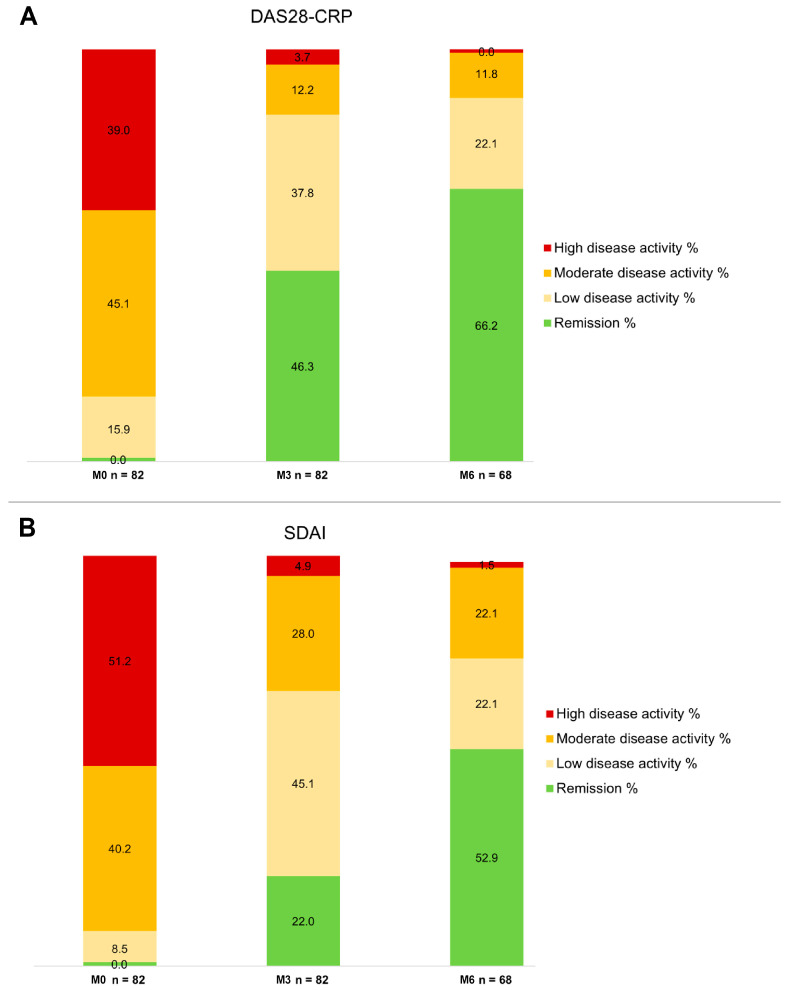
(**A**) Change in DAS28 CRP at follow-up compared to baseline. (**B**) Change in SDAI at follow-up compared to baseline.

A significant proportion of patients obtained DAS28-CRP remission at M3 and M6 (46.3% and 66.2%, respectively) or DAS28-CRP low disease activity (37.8% and 22.1%, respectively) (Table 2, Figure 4). When patients were stratified according to previous exposure to at least one bDMARD, 24 (48%) (vs. 36.8% in naïve patients) of patients at M3 and 27 (64.3%) of patients at M6 (vs. 69.2% in naïve patients) treated with filgotinib reached DAS28-CRP remission; in addition, 36.4% and 50% of the patients already treated with another tsDMARD reached remission at M3 and M6, respectively.

Consequently, only 13 (15.9%) patients at M3 and 8 (11.8%) patients at M6 were classified as having moderate or high disease activity on the basis of DAS28-CRP values.

## 4. Discussion

Filgotinib is the newest molecule of the JAKi class, and what distinguishes this product from other members of the same family is its stronger preferential binding with JAK1. Indeed, while all JAKis have demonstrated a favorable safety profile in RCTs, preclinical data suggest that a higher selectivity for JAK1 may confer additional benefits, especially in preserving hematopoietic and thrombotic homeostasis [21,22,23].

The clinical development program for filgotinib included two phase II RCTs (DARWIN 1, 2, 3) and three phase III RCTs, followed by LTE studies (FINCH 1, 2, 3, 4) [8,9,10,11,12]. These studies consistently demonstrated the efficacy of filgotinib 200 mg in improving signs and symptoms of RA, enhancing physical function, and inhibiting radiographic progression, both in combination therapy and in monotherapy.

In addition, a favorable safety profile was demonstrated, with filgotinib exhibiting the lowest incidence of herpes zoster infection in its class [24,25], and an incidence of cardiovascular, thromboembolic, and neoplastic events lower than that expected in the RA population [26,27,28,29,30].

While RCTs are vital for evaluating the safety and efficacy of novel medications in a highly controlled setting, overly rigid inclusion and exclusion criteria may not adequately address the heterogeneity of vulnerable characteristics that are found in real-world populations [31]. Validating RCTs results requires an analysis of the performance of these molecules in a real population under real-life conditions. To date, real-world reports on filgotinib are limited, primarily because most studies have focused on earlier-generation agents. Tofacitinib, in particular, came under increased scrutiny following the publication of the ORAL Surveillance post-marketing safety trial [13], which failed to demonstrate its non-inferiority compared to TNFi with respect to the risks of MACE and cancers.

In this context, we conducted a retrospective review of clinical data encompassing all patients treated with filgotinib before January 2022, prior to the publication of the results from the ORAL Surveillance trial [13]. Indeed, as a result of these findings, the European Medicines Agency’s (EMA) Committee for Medicinal Products for Human Use (CHMP) issued a set of measures aiming to mitigate the risk of serious side effects that was extended to all members of the JAKi family across all approved indications [20], leading to restrictions in the use of this drug class. Specifically, these restrictions apply to individuals aged 65 years or above, those at an elevated risk of MACE, individuals who smoke or have a prolonged history of smoking, and those at an increased risk of cancer.

Based on data obtained before the release of the EMA recommendations, our cohort provides an authentic snapshot of filgotinib use in real-world clinical practice by a collaborative group of Italian rheumatologists. This cohort, which reflects a pragmatic prescription of filgotinib, included 82 individuals with a high prevalence of the risk factors identified by EMA: 47.6% of individuals were aged 65 years or older, 23.1% were current or former smokers, and 4.9% had a history of ASCVD. Moreover, other traditional cardiovascular risk factors were present in a significant proportion of patients: obesity/overweight in 64.6%, hypertension in 52.4%, dyslipidemia in 34.1%, and type 2 diabetes in 14.6%.

Although these characteristics collectively describe a population at increased risk of MACE, only three patients in our cohort reported adverse events requiring drug discontinuation, none of which was related to the cardiovascular system. One patient with metastatic cancer diagnosed one month after treatment initiation, and thus likely preexisting, discontinued treatment. These findings align seamlessly with the results presented in a recent study named RELIFIRA [32], wherein the authors retrospectively analyzed 120 patients treated with filgotinib. In this study, 54.6% of the population was aged 65 years or older, 7.21% had a history of ASCVD, 15.5% had diabetes, and 47.4% had hypertension.

Concerning effectiveness, it is crucial to note that all patients had previously failed conventional DMARDs, and 61% received at least one bDMARD (22% three or more–up to six–previous bDMARDs); in addition, 11 patients (13.4%) had already been exposed to another JAKi. These characteristics delineate a specific subset of “difficult-to-treat” patients with RA [33]. Despite the challenging clinical scenario, filgotinib demonstrated remarkable efficacy, with only three primary failures. At month 3, 46.3% achieved DAS28-CRP remission, and 37.8% attained low disease activity. At month 6, 66.2% achieved remission, with 21.1% in DAS28-CRP low disease activity. Regarding the SDAI index, at three months, 45.1% exhibited moderate disease activity, with 22% achieving remission. At six months, 22.1% had moderate disease activity, and 52.9% were in remission.

In addition, a significant effect was observed for VAS pain, which decreased at 3 months and remained consistently low at 6 months, and for the number of tender and swollen joints. The benefit in the pain domain is a distinctive feature of JAKi [34], and pooled data from the FIL development program suggest that the analgesic benefit of FIL starts within two weeks and is sustained over time [35].

Cumulatively, these results are consistent with the RELIFIRA study, confirming filgotinib effectiveness in managing difficult-to-treat real-world patients with RA [33] and with a phase III clinical trial in patients with RA with inadequate response to methotrexate [12]. A final notable finding relates to the number of patients treated with filgotinib monotherapy, representing 69.5% of the observed cohort. This percentage underscores that the positive outcomes in terms of effectiveness and safety can be mainly attributed to the effects of filgotinib, despite the challenging clinical context in which both conventional and biological therapies have previously failed.

Our study has several limitations to acknowledge. Specifically, it is a single-arm retrospective observational study, which limits the ability to make direct comparisons with a control group. Additionally, the quality of existing data may not be uniform across centers, potentially affecting the accuracy of our conclusions. Furthermore, the relatively small sample size and the short duration of follow-up may not be sufficient to capture certain aspects, such as cardiovascular risk. Finally, we cannot rule out the presence of confounding variables that may have influenced the observed outcomes.

In conclusion, despite the inherent limitations of a retrospective study design, our data provide additional evidence supporting the effectiveness and safety of filgotinib in a real-world context. The results indicate that treatment with filgotinib led to a significant rate of remission after 6 months, although 61% of patients had previously been exposed to either a bDMARD or another JAKi. Furthermore, only four patients discontinued treatment due to safety concerns, which suggests a favorable safety profile for filgotinib. It is important to note that the majority of patients had at least one of the risk factors identified by the European Medicines Agency (EMA) for limiting the prescription of JAK inhibitors. These findings warrant further investigation to confirm the long-term safety and efficacy of filgotinib in diverse patient populations.

## Figures and Tables

**Table 1:** General characteristics of the study population (n = 82).

**Age, years**	62 ± 13
**Female gender, n (%)**	63 (76.8)
**Disease duration, years**	13 ± 9
**BMI category**	
Underweight, n (%)	4 (4.9)
Normal weight, n (%)	25 (30.5)
Overweight, n (%)	38 (46.3)
Obesity, n (%)	15 (18.3)
**Smoking**	
Current smoker, n (%)	12 (14.6)
Former smoker, n (%)	7 (8.5)
**History of ASCVD, n (%)**	4 (4.9)
**Type 2 diabetes, n (%)**	12 (14.6)
**High blood pressure, n (%)**	43 (52.4)
**Dyslipidemia, n (%)**	28 (34.1)
**RF positive, n (%)**	59 (72.0)
**ACPA positive, n (%)**	50 (61.0)
**bDMARDs failure, n (%)**	50 (61.0)
N° of previous bDMARDs, n [range]	1 [1–6]
Received three or more bDMARDs, n (%)	18 (22)
**JAKi failure, n (%)**	11 (13.4)
**Current treatment with steroids**	
≤5 mg/d prednisone-equivalent, n (%)	34 (41.5)
>5 mg/d prednisone-equivalent, n (%)	10 (12.2)
**FIL monotherapy, n (%)**	57 (69.5)
**MTX combination therapy, n (%)**	24 (29.3)

Data are expressed as the mean ± standard deviation (SD) or number (percentage), as appropriate. Legend: ACPA, anti-citrullinated protein antibodies; ASCVD, atherosclerotic cardiovascular disease; bDMARDs, biologic disease-modifying antirheumatic drugs; BMI, body mass index; FIL, filgotinib; JAKi, Janus kinase inhibitors; MTX, methotrexate; RF, rheumatoid factor.

**Table 2:** Measures of effectiveness during follow-up.

	M0 (n = 82)	M3 (n = 82)	M6 (n = 68)	*p* Value M0 vs. M3	*p* Value M0 vs. M6	*p* Value M3 vs. M6
**VAS pain, mm**	7 ± 2	3 ± 2	2 ± 1	<0.0001	<0.0001	0.05
**Tender joints, n**	8 ± 5	2 ± 3	1 ± 2	<0.0001	<0.0001	0.22
**Swollen joints, n**	5 ± 3	1 ± 1	0 ± 1	<0.0001	<0.0001	>0.99
**DAS28-CRP categories**						
Remission, n (%)	0.0 (0.0)	38 (46.3)	45 (66.2)	<0.0001	<0.0001	0.021
Low disease activity, n (%)	13 (15.9)	31 (37.8)	15 (22.1)	0.002	0.40	0.05
Moderate disease activity, n (%)	37 (45.1)	10 (12.2)	8 (11.8)	<0.0001	<0.0001	>0.99
High disease activity, n (%)	32 (39.0)	3 (3.7)	0 (0.0)	<0.0001	<0.0001	0.25
**SDAI categories**						
Remission, n (%)	0 (0.0)	18 (22.0)	36 (52.9)	<0.0001	<0.001	0.0001
Low disease activity, n (%)	7 (8.5)	37 (45.1)	15 (22.1)	<0.0001	0.022	0.003
Moderate disease activity, n (%)	33 (40.2)	23 (28.0)	15 (22.1)	0.138	0.02	0.45
High disease activity, n (%)	42 (51.2)	4 (4.9)	1 (1.5)	<0.0001	<0.0001	0.38

Data are expressed as the mean ± standard deviation (SD) or number (percentage), as appropriate. The Kruskal–Wallis H test was used to compare continuous variables; Fisher’s exact test was used to compare dichotomous variables. Legend: DAS28-CRP, disease activity score including 28 joints; SDAI, simplified disease activity index; VAS, visual analog scale.

## Data Availability

The raw data supporting the conclusions of this article will be made available by the authors upon request.
